# Closed‐Loop Wearable Device Network of Intrinsically‐Controlled, Bilateral Coordinated Functional Electrical Stimulation for Stroke

**DOI:** 10.1002/advs.202304763

**Published:** 2024-03-01

**Authors:** Shuxing Xu, Chengyu Li, Conghui Wei, Xinfang Kang, Sheng Shu, Guanlin Liu, Zijie Xu, Mengdi Han, Jun Luo, Wei Tang

**Affiliations:** ^1^ Beijing Institute of Nanoenergy and Nanosystems Chinese Academy of Sciences Beijing 101400 China; ^2^ Center on Nanoenergy Research School of Physical Science & Technology Guangxi University Nanning 530004 China; ^3^ School of Nanoscience and Technology University of Chinese Academy of Sciences Beijing 100049 China; ^4^ Rehabilitation Medicine Department The Second Affiliated Hospital of Nanchang University Nanchang City 330006 P. R. China; ^5^ Department of Biomedical Engineering College of Future Technology Peking University Beijing 100871 China; ^6^ Institute of Applied Nanotechnology Jiaxing Zhejiang 314031 China

**Keywords:** closed‐loop network, functional electrical stimulation, stroke, wearable devices

## Abstract

Innovative functional electrical stimulation has demonstrated effectiveness in enhancing daily walking and rehabilitating stroke patients with foot drop. However, its lack of precision in stimulating timing, individual adaptivity, and bilateral symmetry, resulted in diminished clinical efficacy. Therefore, a closed‐loop wearable device network of intrinsically controlled functional electrical stimulation (CI‐FES) system is proposed, which utilizes the personal surface myoelectricity, derived from the intrinsic neuro signal, as the switch to activate/deactivate the stimulation on the affected side. Simultaneously, it decodes the myoelectricity signal of the patient's healthy side to adjust the stimulation intensity, forming an intrinsically controlled loop with the inertial measurement units. With CI‐FES assistance, patients’ walking ability significantly improved, evidenced by the shift in ankle joint angle mean and variance from 105.53° and 28.84 to 102.81° and 17.71, and the oxyhemoglobin concentration tested by the functional near‐infrared spectroscopy. In long‐term CI‐FES‐assisted clinical testing, the discriminability in machine learning classification between patients and healthy individuals gradually decreased from 100% to 92.5%, suggesting a remarkable recovery tendency, further substantiated by performance on the functional movement scales. The developed CI‐FES system is crucial for contralateral‐hemiplegic stroke recovery, paving the way for future closed‐loop stimulation systems in stroke rehabilitation is anticipated.

## Graphical abstract

This closed‐loop wearable device network, which incorporates intrinsically‐controlled, bilateral coordinated functional electrical stimulation, utilizes electromyographic signals from the rectus femoris and tibialis anterior muscles for activation and modulation of functional electrical stimulation, aims to aid walking at ankle joint angles, alleviates oxyhemoglobin burden, and enhance motor function during clinical interventions for stroke‐induced foot drop patients.

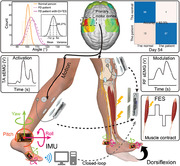

## 1 Introduction

Stroke is a global health problem that is common, serious, and disabling, consistently reported as one of the most common causes of death and the leading cause of adult disability.^[^ ^1^, ^2^, ^3^ ^]^ According to the statistics of the World Health Organization, over 5 million lives are lost annually, and more than 15 million people experience a significant loss of normal life due to stroke.^[^ ^4^, ^5^ ^]^ Among them, damage to the motor cortex or corticospinal tract often results in contralateral hemiplegia with significant persistent distal weakness.^[^ ^6^, ^7^ ^]^ Individuals with this weakness pattern frequently struggle to actively dorsiflex the foot during the swing phase of gait, which is referred to as foot drop (FD).^[^ ^8^, ^9^, ^10^ ^]^ The abnormal gait caused by foot drop significantly increases the risk of falls, threatening patients’ safety and health, and resulting in long‐term consequences for patients and their families.^[^ ^11^ ^]^

Functional electrical stimulation (FES) stands out as an emerging wearable technology by uses low‐frequency electrical pulses^[^ ^12^ ^]^ to stimulate the tibialis anterior (TA) and lateral common fibular nerves on the lower leg,^[^ ^13^ ^]^ driving the dorsiflexion of the ankle joint, and realizes the walking assistance.^[^ ^14^, ^15^, ^16^ ^]^ FES addresses issues associated with conventional treatment of ankle foot orthosis,^[^ ^8^, ^9^, ^17^ ^]^ including the potential development of contracture, difficulty in standing up, and discomfort. Moreover, FES also has a positive effect on reconstructing motor nerve function.^[^ ^15^, ^18^, ^19^ ^]^ However, traditional FES is switched by an inclination sensor installed on the leg, or pressure sensors on the sole,^[^ ^20^, ^21^, ^22^ ^]^ which is passive and post‐conscious controlling (Table S1, Supporting Information). Additionally, the intensity of stimulation lacks individual and real‐time adjustability,^[^ ^23^, ^24^ ^]^ which might lead to reduced clinical efficacy, or symptoms such as muscle fatigue and soreness.^[^ ^25^ ^]^ Some prior studies mentioned using surface electromyography (sEMG) signals to characterize muscle activities,^[^ ^26^, ^27^ ^]^ attempting to use them for adjusting the stimulation intensity.^[^ ^28^, ^29^ ^]^ However, the stimulation is simply preset before patients’ practical use, lacking continuous adjustment based on real‐time movements,^[^ ^30^ ^]^ which can result in abnormal gait stiffness and uncoordinated body behaviors.

Here, we developed a closed‐loop and intrinsically‐controlled functional electrical stimulation (CI‐FES) system, utilizing the intrinsic ipsilateral rectus femoris (RF) sEMG signal to switch the stimulation applied on the affected TA muscle, and employing the contralateral healthy TA muscle's sEMG signal to real‐time modulate the stimulation intensity, so as to realize a bilateral coordinated walking action for patients. High‐precision inertial measurement units (IMUs) engineered in a wireless, wearable format, were used to test physical indicators such as ankle angle, angular velocity, and gait duration, and were employed to adjust the multiple of sEMG signals to regulate FES. Functional near‐infrared spectroscopy (fNIRS) was utilized to characterize neural activity in the brain, including oxyhemoglobin (HbO), deoxyhemoglobin (HbR), and total hemoglobin (HbT). Following the assessment of physical and physiological indicators in 15 patients utilizing CI‐FES, it was observed that the ankle joint angle on the affected side significantly approached the unaffected side, and a substantial reduction and balance in HbO consumption within the primary motor cortex area, collectively indicating a significant trend toward assistance. Moreover, over the 54 days of treatment, the distinction between patients and normal subjects gradually diminished through machine learning analysis, and a notable enhancement in motor performance was evident in the Fugl‐Meyer assessment (FMA), Brunnstrom stage, and Ashworth scale. All these collective findings underscore the considerable significance of the CI‐FES system in assisting and rehabilitation of FD stroke patients.

## 2 Results and Discussion

### 2.1 Design of the CI‐FES System

**Figure 1:**
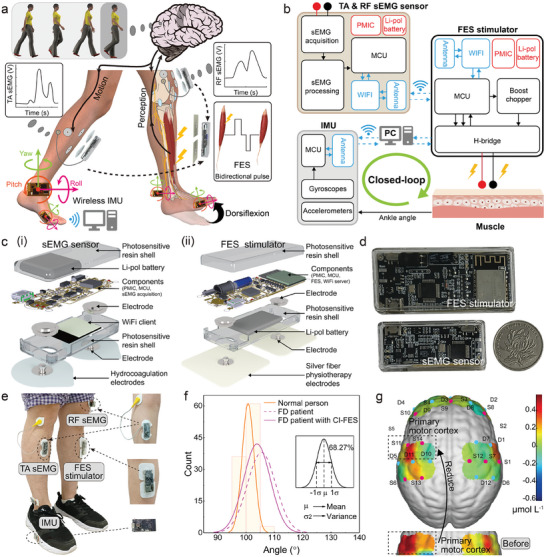
Design of the CI‐FES system. a) Illustration of the CI‐FES system comprising three main components: a RF sEMG sensor (on‐off switch) on the affected side, a TA sEMG sensor (intensity modulator) on the healthy side, and a FES stimulator on the affected TA muscles. Walking actions are monitored by 4 IMUs on the upper ankles and feet. b) Schematic diagram of the closed‐loop FES stimulation, using RF sEMG, TA sEMG, and IMU feedback. c) Exploded view of the i) sEMG sensor and ii) FES stimulator. d) Images of the sEMG sensor and FES stimulator. e) A FD patient wearing the CI‐FES system. f) The CI‐FES system modifies the patient's walking gait, resulting the patient's actions closer to those of a normal individual in both mean value and variance. The maximum plantarflexion, associated with lifting the heel from the ground to pointing the toes downward, is collected (100 steps), counted, analyzed, and plotted in the distribution statistics histogram at 5° intervals. The distribution curve, derived from the statistical histogram, illustrates the centralization of gait through the mean value and the central tendency through the variance. g) In the task state with CI‐FES, HbO concentration in the primary motor cortex (areas marked with dotted lines) on the affected side is significantly lower than without wearing it.

The CI‐FES consists of three components (**Figure** **1**a), an RF sEMG sensor, detecting sEMG signals at the RF muscle for toggling stimulation on/off; a TA sEMG sensor, collecting sEMG signals at the unaffected TA muscle to modulate the stimulation intensity; and a FES stimulator, generating adjustable electric pulses. When individuals attempt to walk, the brain's primary motor cortex sends nerve signals to the target muscle, resulting in the generation of motor unit action potentials^[^ ^31^ ^]^ in the muscle fibers. These motor unit action potentials are recorded by the wearable sEMG sensor (Figure 1a insert of TA sEMG and RF sEMG) and transmitted to the FES stimulator via WiFi. The FES stimulator analyzes the received data to generate stimulation modulated by RF timing and contralateral TA sEMG waveform, causing the affected TA muscle contract (Figure 1a insert and Figure S2, Supporting Information), facilitating proper dorsiflexion of the ankle joint. Furthermore, the dynamic behavior of both feet is captured by four attached IMUs,^[^ ^26^, ^32^ ^]^ which are then used for closed‐loop feedback and adjustment^[^ ^33^, ^34^, ^35^ ^]^ on the electric stimulation to attain output within a specified range (Figure 1b and Figure S3, Supporting Information). The sEMG sensors (Figure 1c(i)) are affixed to the RF muscle on the affected side and the TA muscle on the healthy side (Figure S4, Supporting Information).^[^ ^36^, ^37^ ^]^ A wireless chip is employed to transmit the sEMG data to the FES stimulator (Figure 1c(ii)) that is attached to the affected TA muscle. Consequently, the stimulator activates electrical stimulation based on the RF sEMG and adjusts stimulation amplitude based on the TA sEMG and IMUs. The stimulation signal utilizes a bidirectional pulse‐width modulation (PWM) wave, and is delivered to the skin through hydrogel electrodes^[^ ^27^ ^]^ with silver nanofibers as substrate (Figure S5, Supporting Information). Images of the sEMG sensor and FES stimulator are depicted in Figure 1d and Figure S6 (Supporting Information). A patient‐wearing photo is shown in Figure 1e (Note S1, Supporting Information). Figure 1f illustrates the statistical distribution of a patient's maximum plantarflexion after wearing CI‐FES, monitored by IMUs, indicating a tendency to approach that of normal subjects, thus verifying the CI‐FES's effectiveness. Moreover, with the assistance of CI‐FES, the HbO concentration^[^ ^38^ ^]^ of the affected side decreases (Figure 1g) in the task state, which implies the CI‐FES makes patients’ activity more labor‐saving.

### 2.2 Intrinsic Switch by sEMG Signal

**Figure 2:**
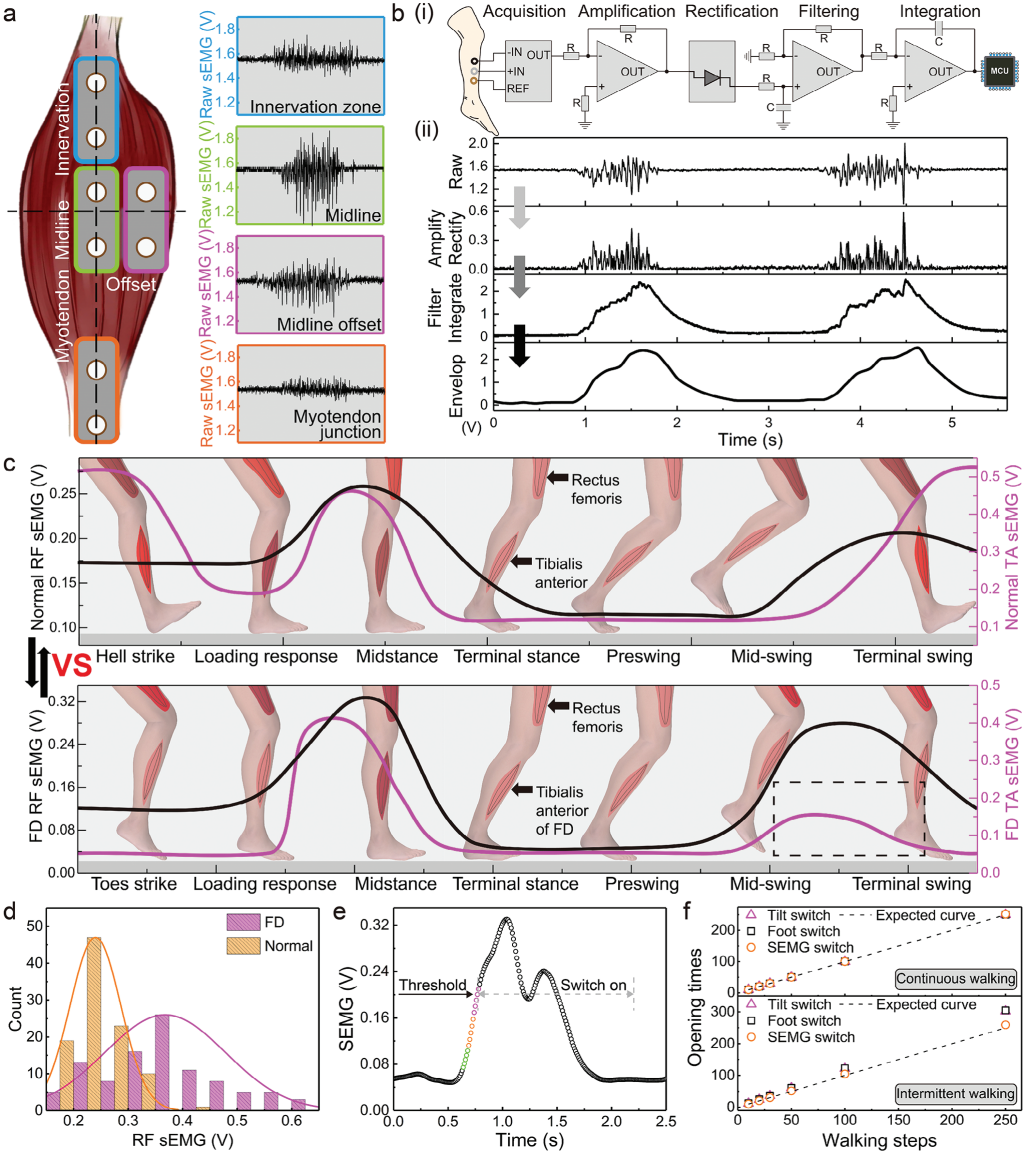
Intrinsic switch by sEMG signal. a) While the subject was seated with lower limbs hanging freely and relaxed, the raw sEMG signals from various areas of the TA muscle on the healthy side were recorded. b) i) Processing of raw sEMG signal, involving amplification, rectification, filtering, and integration. ii) Transformation of the waveform from multi‐peak to a smooth curve during intentional dorsiflexion of the TA muscle in a sitting position with a suspended leg, displayed on a computer after using a nonlinear envelope algorithm. c) SEMG signals of the RF and TA muscles in one gait cycle, with upper and lower figures showing normal subject and FD patient data, respectively. d) Statistic analysis of RF sEMG signals from the normal subject and FD patient. e) Stimulus‐on principle: the moving average of the measured sEMG signal is calculated, and compared to the threshold to determine FES turn‐on time. Stimulation persists beyond the threshold, guided by a countdown time set by the program. f) Switching performance comparison using sEMG sensor, tilt sensor, and foot pressure sensor as the switch, during continuous and intermittent walking for 10, 20, 30, 50, 100, and 250 steps. The expected curve represents an accuracy of 100%.

The sEMG signals are obtained by two electrodes operating in differential mode, as shown in Figure 1c(i). We placed them in four different locations on the unaffected TA muscle and checked the output difference during the same movement (**Figure** **2**a). It was observed that the one placed in the middle of the muscle output the largest value. Subsequently, the raw sEMG signals^[^ ^39^ ^]^ underwent processing (including amplification, rectification, and integration, as shown in Figure 2b), were wirelessly transmitted to the computer, and displayed after nonlinear envelope processing (Figure S7, Movie S1, Supporting Information).

During walking, successive activities of hips, knees, ankles, and toes are generated.^[^ ^40^ ^]^ In normal subjects, gait is characterized by rhythmical, periodic, and stable patterns, dividing into a stance phase and a swing phase.^[^ ^16^, ^39^ ^]^ While that of FD patients is different.^[^ ^41^, ^42^ ^]^ Upon comparing the sEMG signals of the RF and TA muscles (enveloped sEMG phase‐domain signal shown in Figure 2c, and the unenveloped time‐domain signal in Figure S8, Supporting Information), it was observed that FD patients exhibit RF sEMG similar to that of normal subjects, with peaks in both stance and swing phases, indicating their ability to lift their legs.^[^ ^26^, ^43^, ^44^ ^]^ Nevertheless, they often land on their toes after swinging (normal subjects commonly land on heels), as verified by the lower sEMG signal of the TA (dotted line in Figure 2c). Statistical results from repeated measurements (Figure 2d) show that the RF sEMG of FD patients is comparable to, or even larger than that of normal subjects. This is because patients intentionally raise their sagging feet, causing the muscles to work even harder than usual. Consequently, we selected the ipsilateral RF sEMG as the intrinsic switch for FES (Figure 2e).^[^ ^45^, ^46^, ^47^ ^]^ Compared with the insole pressure sensor and the inclination sensor (Figure S9 and Note S2, Supporting Information), the sEMG switch demonstrated stability during long‐term continuous walking, intermittent walking (Figure 2f), and squatting (Figures S10 and S11, Supporting Information). Notably, since the sEMG signal originates from neurological activity, the switch is intrinsically controlled, offering greater accuracy than the inclination switch and avoiding false mechanical triggers caused by a slight tilt,^[^ ^20^ ^]^ while also being more convenient than the foot pressure switch, which requires the patient has to wear special shoes.

### 2.3 Adjustable FES and its Performance

**Figure 3:**
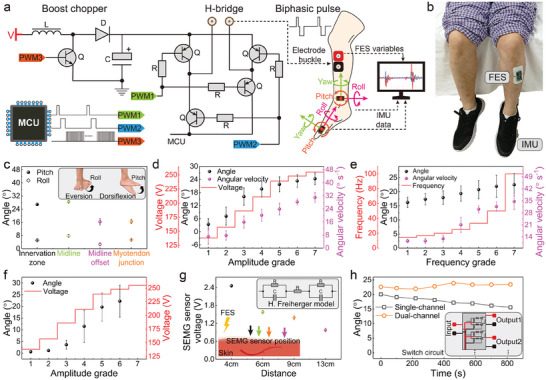
Adjustable FES and its performance. a) Circuit diagram of the adjustable FES stimulator. The MCU generates three PWM waves. PWM1 and PWM2 are adjusted and delivered to the muscle, and PWM3, a high‐frequency pulse determines the stimulation intensity. b) Photograph of subjects in a seated position during the adjustable‐FES test. c) Ankle bending, resulting from FES in different locations of the affected TA muscle when employing the position illustrated in Figure (b). Effect of FES with different intensities d) and frequencies e) on the ankle bending angle and angular velocity. f) Patients’ response to different intensities of FES. Values in (d) to (f) are averaged test results from six persons, with error bars representing standard deviation. g) Influences on sEMG signals caused by FES at different distances. The inset shows the resistance‐capacitance model of the human body. h) Subjects’ dorsiflexion angles in the muscle fatigue test under single‐ and dual‐channel FES. Stimulation in different muscle sites and timings effectively relieves the decrease in dorsiflexion angle and muscle fatigue. Inset is the schematic diagram of the multi‐channel circuit.

To administer personalized electric pulses for FES, we engineered an adjustable FES stimulator, as shown in **Figure** **3**a. Three PWM waves (PWM1, PWM2, and PWM3) modulate both the Boost chopper circuit and the H‐bridge circuit (Note S3, Supporting Information). The PWM3 in Figure 3a (frequency: 16.67 KHz, duty cycle: 83.3%) is a high‐frequency switching pulse, which determines the stimulation intensity by controlling the on‐off state of a triode. The PWM1 and PWM2, generated by the microprocessor (MCU), traverse the H‐bridge circuit to convert the original unidirectional pulses into bidirectional pulses, then amplified to a certain value by the Boost chopper circuit, and delivered out through a hydrogel electrode. The wearers’ actions are monitored by IMUs attached to ankles and soles (Figure S12, Table S3, and Note S4, Supporting Information). A sitting position with the foot hanging over (Figure 3b) was adopted to check the ankle bending under various stimulations. Figure 3c illustrates the variation in bending angle as the FES's location changes on the affected TA muscle, suggesting that stimulation in the middle area induces significant contraction. Subsequently, we systematically employed the method of controlling variables to apply seven grades of stimulation intensity, frequency, pulse width, and pulse multiples to six healthy participants and resented the results about their ankle bending angle and velocity (discrepancy and mean values during dorsiflexion, the processes are illustrated in Figures S13–S15 and Note S4, Supporting Information) in Figure 3d,e and Figure S16–S19. In the intensity test, we incrementally increased the number of PWM3 (Figure 3a) from 20 to 140 in a step of 20, causing the pulse voltage to gradually rise from 138 to 255 V (Figure S16, Supporting Information). The ankle bending angle and angular velocity exhibited a slight incline followed by saturation (Figure 3d; Movie S2, Supporting Information). Considering the muscle's fragility and tolerance, we capped the maximum stimulation voltage at 240 V.^[^ ^13^, ^48^ ^]^ Then the influence of stimulation frequency was examined and plotted in Figure 3e. As the frequency increased (Figure S17, Supporting Information), the angle and angular velocity showed a slight upward trend. Other influencing factors, such as pulse width, and pulse polarity, were studied and presented in Figures S18 and S19 (Supporting Information).

For comparison, we conducted the intensity test on six patients (Figure 3f), showing that the bending behavior of the FD patient under different stimulation intensities is similar to that of healthy individuals, implying the feasibility of adjustable FES. Therefore, we set the stimulating frequency at 33 Hz, the pulse width at 200 us, the upper and lower pulse width ratio at 1:2, and the intensity modulated via the contralateral TA sEMG. Figure 3g illustrates the impact of acquiring sEMG sensors during FES application to receptors’ resting lower limb, demonstrating a gradual decrease in the measured sEMG values with increasing distance, ensuring concurrent operation of the FES and sEMG sensor as long as the distance is sufficiently large. Since muscle contraction is energy‐consuming, and the fatigue often occurs after prolonged repetitive stimulating,^[^ ^49^, ^50^ ^]^ we subjected patients to 900 s of repetitive FES, observing a gradual diminishment in their response to the same electrical stimulation due to muscle fatigue (black line in Figure 3h). Hence, multi‐channel FES was developed (Figure 3h inset and Figure S20, Supporting Information).^[^ ^19^ ^]^ Taking a two‐channel FES circuit as an example, electrical pulses are delivered alternately through two channels, stimulating different sites and timings in the same muscle, and thus minimizing fatigue induced by continuous stimulation. As a comparison, the two‐channel stimulation effectively slows down the decrease in ankle bending angle caused by muscle fatigue (Figure 3h; Figure S21, Supporting Information).

### 2.4 Demonstrations of the Closed‐loop and Intrinsically‐Controlled FES System

**Figure 4:**
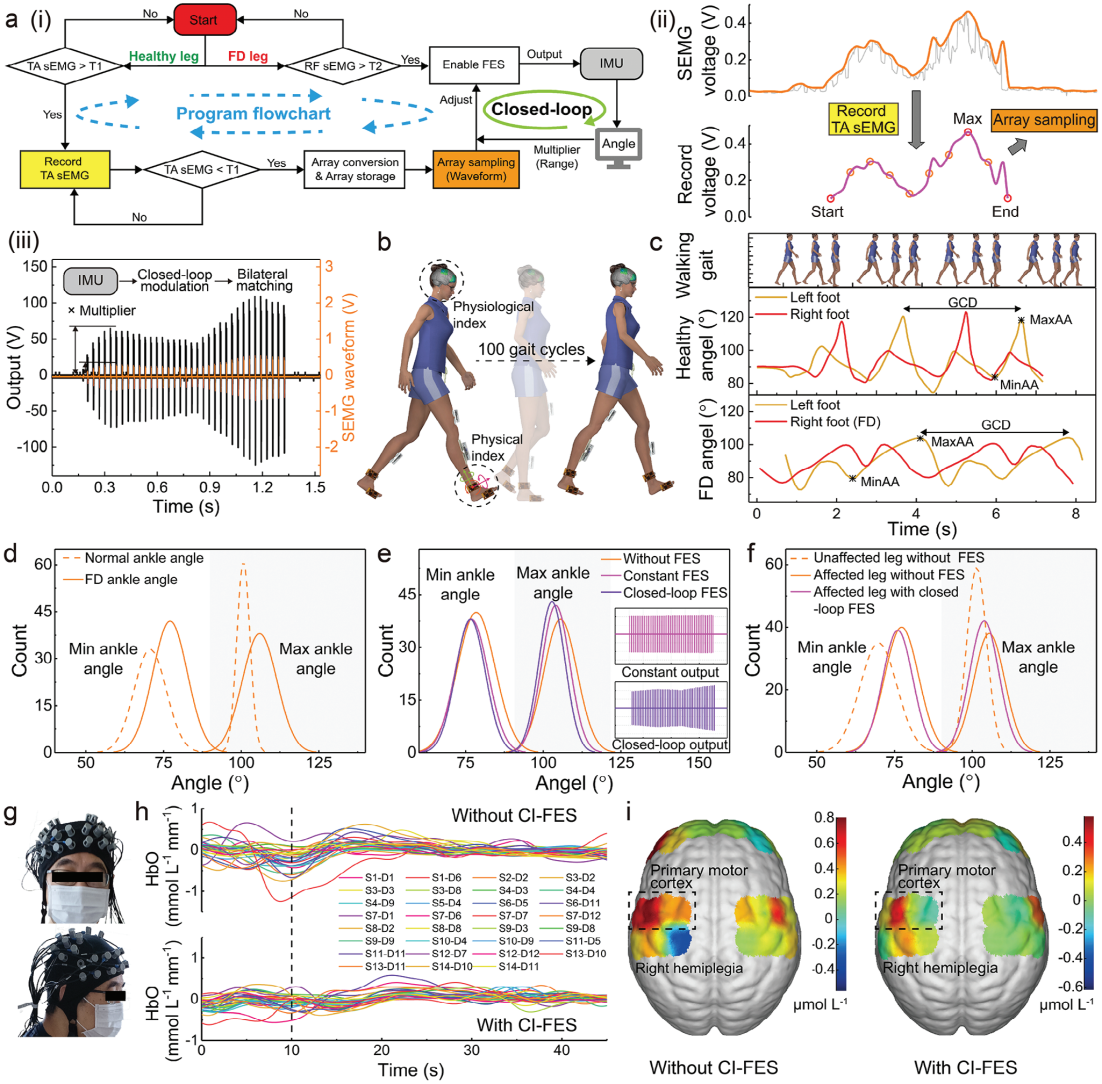
Demonstration of the closed‐loop and intrinsically‐controlled FES system. a) i) Workflow diagram of CI‐FES. ii) Recording and array sampling of the TA sEMG signal. iii) Stimulation output following waveform/range modulation by sEMG signals and IMUs angles. b) Schematic diagram for monitoring physical and physiological indicators. c) Gait patterns of a normal subject and a patient recorded by the IMUs. The maximum and minimum ankle angle values, along with the gait cycle duration, are marked by MaxAA, MinAA, and GCD. Together with the maximum angular velocity, these values constitute the feature set for data analysis. d) Differences in the distribution of MaxAA and MinAA in patients and normal gait. e) Patients’ gait variation, with no/constant/closed‐loop‐controlled FES. Insets are the oscilloscope waveforms of FES. f) Comparison of gait on both sides of the FD patient wearing CI‐FES. Affected leg's ankle bending angles approach unaffected legs under the stimulation of CI‐FES. g) Photographs of FD patients wearing fNIRS. h) 31‐channel cerebral HbO curve of FD patients wearing and not wearing CI‐FES while performing fixed movements. i) Oxyhemoglobin value in (h) is mapped to a 3D map of the brain. The area marked by the dotted box in (i) is the primary motor cortex related to body movement. Patient with right hemiplegia shows significant changes in HbO concentration (max reduced from 0.8 to 0.52 µmol L^−1^) in the left primary motor cortex with and without CI‐FES.

**Figure** **4**a(i) shows the workflow of the CI‐FES system. When the patient's healthy side moves and the TA sEMG exceeds a threshold1 (T1 = 0.15 V), the signal is recorded (Figure 4a(ii)) by the MCU until it drops below T1. Subsequently, the recorded TA sEMG single undergoes sampling and storage for intensity modulation (Note S5, Supporting Information). When the patient's affected side moves and the RF sEMG surpasses a threshold2 (T2 = T1), the FES is activated, delivering electric stimulation (Figure 4a(iii)) adjusted by the acquired TA sEMG of the healthy side and the IMUs feedback to ensure bilateral coordinated movement. Upon completion of modulation, the space in the storage array is released to accommodate the signal generated by the next gait and start the above flow again. Among them, the feedback from IMU angles is employed to closed‐loop adjust the output amplitude through the multiplier burned by the host computer,^[^ ^35^ ^]^ in conjunction with the sEMG signal on the contralateral side, enabling the FES output to fluctuate within the range explored in Figure 3d. Through the physical indicators measured by IMUs (Figure S22, Supporting Information) worn on the ankles and soles of the feet,^[^ ^32^ ^]^ along with physiological indicators measured by the fNIRS worn on the head, we can analyze the assistive functionality of the CI‐FES system (Figure 4b). Figure 4c shows that, both for patients and healthy individuals, there exists a maximum ankle angle (MaxAA) and a minimum ankle angle (MinAA) in one cycle of walking. The time between two adjacent MaxAAs is defined as the gait cycle duration (GCD). Parameters, including MaxAA, MinAA, maximum angular velocity, and GCD of the left foot and right foot serve as feature values to discern differences between FD patients and healthy individuals. Specifically, the MaxAA and MinAA, extracted from 100 steps of healthy individuals and FD patients, are counted (Figure S23, Supporting Information) and plotted in Figure 4d and Figure S24 (Supporting Information). It is evident that both the MaxAA and MinAA of normal individuals are more concentrated compared to those of patients. Furthermore, the mean value of the MaxAA of healthy individuals is smaller than that of patients, ranging from 100.72° to 105.93°, which might be attributed to the uncontrollable actions of patients. Subsequently, FES is applied to patients’ affected side (Figure 4e and Figure S25, Supporting Information), revealing that closed‐loop‐controlled FES resulted in a reduction in both the mean value and variance of MaxAA, decreasing from 105.53° and 28.84 to 102.81° and 17.71, respectively, compared to that under no/constant (240 V, 33 Hz, pulse width 200 us and 1:2) FES, indicating improved controllability of patients’ ankle joint. Additionally, the maximum ankle angular velocity and GCD tend to be faster and shorter, respectively.^[^ ^8^, ^39^ ^]^ Further comparison of the patient's two sides under CI‐FES (Figure 4f and Figure S26, Supporting Information) shows that the MaxAA of the affected side notably changed (mean value and variance shift of 1.7° and 2.83, respectively) under closed‐loop‐controlled FES, indicating a convergence toward the unaffected side (Movie S3, Supporting Information).

Considering that FES will not only induce changes in physical activities but also cause physiological changes, we employed a non‐invasive, portable fNIRS to detect oxygenation in the brain of FD patients (Figure 4g and Figure S27, Supporting Information).^[^ ^51^, ^52^ ^]^ We examined the concentration of HbO, HbR, and HbT in the frontal lobe, primary motor cortex, and primary somatosensory cortex (Figure 1a insert). During the test, patients followed instructions to walk a certain distance and then rested for over 15 min before the next trial. Data processing details are outlined in the method. Figure 4h shows the change in HbO, directly correlated with oxygenation, reflecting the degree of cerebral region activation level binding with and without CI‐FES under the above exercise. It can be found that the HbO variation of patients with CI‐FES remained at a lower level compared to that without CI‐FES, ranging from −1.39 to 0.68 mmol L^−1^ mm^−1^ and from −0.73 to 0.63 mmol L^−1^ mm^−1^, respectively. We mapped the data representing moments of significant difference during movement onto a 3D brain model (Figure 4i and Figure S28, Supporting Information) to visually compare cerebral HbO consumption and the burden of activation in the left primary motor cortex of the right hemiplegia patient. Observations revealed that upon wearing the CI‐FES, the patient's HbO in the left primary motor cortex significantly decreased from 0.8 to 0.52 µmol L^−1^ at this moment, and a concurrent reduction in the average variance of the HbO curve from 0.019 to 0.015 within ≈45 s of task time,^[^ ^38^ ^]^ indicating CI‐FES's effective assistance (Movie S4, Supporting Information).

### 2.5 Rehabilitation by CI‐FES

**Figure 5:**
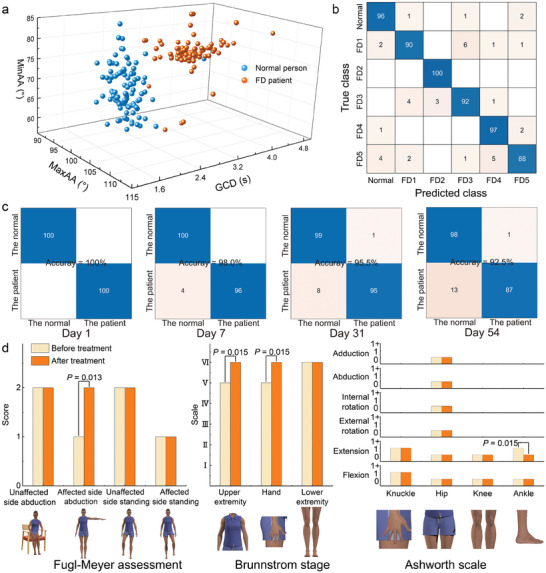
Rehabilitation by CI‐FES. a) Scatterplot distribution of MaxAA, MinAA, and GCD in patients and normal individuals. b) Verification of sample recognition (one normal person and five patients) using the machine learning model. c) Confusion matrix for 54‐day rehabilitation of the patient wearing CI‐FES. Training accuracy between patients and normal subjects under 8‐D features decreased from 100% on the first day, 98% on the 7th day, 95.5% on the 31st day, to 92.5% on the 54th day. d) Changes in FMA (unsupported sitting, unaffected side abduction, affected side abduction, supported standing, unsupported standing, unaffected side stand, affected side stand), Brunnstrom stage (I flaccidity, II spasticity appears, III increased spasticity, IV decreased spasticity, V spasticity continues to decrease, VI spasticity disappears and coordination reappears, VII normal function returns) and improved Ashworth scale (extension, external rotation, internal rotation, abduction, and adduction of shoulder joint, elbow joint, wrist joint, finger joint, hip joint, knee joint, ankle joint) in the FD patient wearing CI‐FES system before and after treatment. The data presented reflects the mode, given the absence of intermediate levels in those scales, with a sample size of *n* = 4. A two‐way analysis of variance (ANOVA) was utilized to statistically examine the significant influence of two factors (pre‐treatment and post‐treatment) on scale assessments.

As both muscles and nerves can be stimulated by CI‐FES, contributing to damage repair in the cerebral cortex and subsequent motor function recovery,^[^ ^15^, ^53^, ^54^ ^]^ we conducted a thorough examination of patients’ rehabilitation. A supervised machine learning training model was employed,^[^ ^27^ ^]^ utilizing eight parameters including MaxAA, MinAA, maximum angular velocity, and GCD, as feature values. In **Figure** **5**a, an exemplified distribution of the patient and healthy individuals is depicted, with MaxAA on the X‐axis, gait time on the Y‐axis, and MinAA on the Z‐axis. As more parameters are incorporated, the distributions of patients and healthy individuals become further separated.^[^ ^55^ ^]^ After a meticulous examination of various training models and kernel functions, we determined that the support vector machine (SVM) models with the median Gaussian kernel function^[^ ^56^ ^]^ outperformed others (Figures S30 and S31, Supporting Information). Subsequently, we selected 600 samples from five patients and one normal subject for training and ten‐fold cross‐validation. The identification accuracy can reach 93.3%, and both the confusion matrix (Figure 5b) and receiver operating characteristic curve (Figure S32, Supporting Information) demonstrated excellent stability. We then employed the machine learning model to evaluate a patient's rehabilitation process during a 54‐day CI‐FES treatment (Figure 5c and Figure S33, Supporting Information). On the first day, the patient was identified correctly with an efficiency of 100%; on the 7th day, the efficiency dropped to 98%, indicating a gradual confusion of features between the patient and the normal subject; on the 31st day and the 54th day, the recognition efficiency further descended to 95.5% and 92.5%. This implies that the characteristics of the patient are gradually becoming similar to those of a normal subject, meaning the CI‐FES has a significant therapeutic effect on the FD patient.

Additionally, a standardized medical test, including FMA (Unsupported sit, unaffected/affected side abduction, stand with/without support, unaffected/affected side standing. The higher the score, the better the performance. Table S4, Supporting Information), Brunnstrom stage (Muscle strength in stroke recovery, Table S5, Supporting Information) and improved Ashworth scale (Resistance during flexion, extension, external/internal rotation, abduction, adduction. The smaller the value, the more normal. Table S6 and Note S6, Supporting Information),^[^ ^57^ ^]^ was chosen to evaluate the patient's physical function before and after treatment with CI‐FES for ≈1.5 months (Figure 5d). The patient, after being treated with CI‐FES, exhibited better performance compared to the one without CI‐FES treatment (Figure S34, Supporting Information). For instance, the extension of the affected side has essentially returned to normal (progressing from the first level to the second level in FMA); the muscle strength of the upper limb and hand has been improved (Brunnstrom stage); the stiffness of the ankle joint has been reduced (ankle extension in Ashworth scale); and the motor function recovered faster (*p*‐value < 0.05).

## 3 Conclusion

Stroke is a widespread and severe cause of disability, often manifesting with foot drop as a characteristic symptom. While functional electrical stimulation has demonstrated notable efficacy in assisting patients with daily walking, challenges such as inaccurate stimulation timing, unadjustable stimulation intensity, and a lack of bilateral symmetry persist. In response, we proposed a closed‐loop wearable device network featuring an intrinsically controlled and bilateral coordinated functional electrical stimulation system. Key advances involve the utilization of intrinsic neural signals in the form of myoelectricity as the stimulation switch, ensuring precise stimulation timing and contributing to further neural restoration; stimulation intensity is modulated through the patient's healthy‐side sEMG, facilitating bilateral coordinated walking actions. Clinical tests conducted on 15 patients revealed that patients’ walking patterns become more regular, gradually resembling those of normal individuals in ankle bending angle, angular velocity, and gait duration. Functional near‐infrared spectroscopy demonstrates that CI‐FES effectively reduces oxyhemoglobin consumption in the primary motor cortex, alleviating the cognitive load during walking actions. Moreover, long‐term treatment with CI‐FES, along with machine learning models, FMA, Brunnstrom stage, and Ashworth scale tests, all indicate significant recovery in FD patients’ motor function. Despite some existing issues, such as insufficient system integration, closed‐loop adjustment parameters, and myoelectric thresholds requiring modification after experimental measurement, we anticipate that the closed‐loop network of intrinsically controlled FES systems is crucial for the recovery of contralateral‐hemiplegia stroke and serves as a pioneering approach for closed‐loop stimulation systems in stroke rehabilitation.

## 4 Experimental Section

### Design, Programming, and Implementation of the CI‐FES System

In sEMG acquisition system, the signal transmitted by the two electrodes undergoes analyzed and modulated by the reference electrode voltage in the AD8236 chip, producing the raw sEMG signal. Following rectification, filtering, integrating, and amplification circuits composed of operational amplifiers in AD8646 and AD8648 chips, the raw sEMG signal was converted to a voltage in the region of 0–3.3 V (Figure 2b). This voltage was then input to the ADC acquisition port of the STM32F103 chip. The MCU converts the analog voltage into a digital signal and transmits the data via the ESP8266‐12S WiFi chip to the server of the FES stimulator for analysis and processing.

The FES stimulator's internal ESP8266‐12S chip acts as a server to receive data transmitted from multiple sEMG sensors in real time. The received data was subjected to threshold judgment and analysis in the STM32F103 chip, and its GPIO port will send out corresponding PWM waves to activate the Boost chopper circuit and modulate the H‐bridge circuit (Figure 3a). Furthermore, the magnification after IMU conversion will be burned into the FES stimulator through the PC. The output of the FES stimulator acts on the TA muscle through the physiotherapy electrodes with silver fiber substrate (Figure 1c(ii)), and can also be measured by an oscilloscope (DSOX2014A produced by KEYSIGHT, Bandwidth 200 MHz, sampling rate 2 GSa s^−1^).

The program of the STM32F103 chip was collaboratively developed by KEIL5 and STM32CubeMX. The firmware burning software for the wireless communication chip was ESP FLASH TOOL. The host computer receiving software was programmable SerialPlot.

The shells of the sEMG sensor and FES stimulator were high‐precision 3D printed products (drawn by SOLIDWORKS software and made by an Objet30 Prime 3D printer), and the material was transparent photosensitive resin. The connectors of the circuit board (processed by JCL company), hydrocoagulation electrodes, or physiotherapy electrodes were all nickel electrodes plated with silver chloride, which was an electrode buckle with an outer diameter of 12 mm and an inner diameter of 3.9 mm. The welded electrode button can be connected to the protruding sub‐button on the patch, facilitating the bidirectional transmission of electrical pulses or myoelectric signals. The hydrogel's robust adhesion obviates the necessity for additional bandages, enhancing the device's portability. Moreover, the high conductivity of the hydrogel prevents signal distortion, and its softness facilitates long‐term wear.

### Participant Recruitment and Testing

All tests adhered to international standards obtained ethical authorization from the International Committee of Medical Journal Editors (Authorization No. ChiCTR2200065112), and received approvals from The Second Affiliated Hospital of Nanchang University and Beijing Institute of Nanoenergy and Nanosystems, Chinese Academy of Sciences. The participants, comprising 15 patients with foot drop for testing and treatment (some of whom are bedridden, as indicated in Table S2, Supporting Information), and six normal subjects as test criteria, were informed about the experimental procedure and provided signed informed consent forms before participation.

During testing of electrical stimulation parameters, both patients and normal subjects (sample sizes were six respectively) adopted a sitting position with feet hanging over (Figure 3b), keeping the lower body relaxed without free movement. The skin at the electrodes was wiped with a wet tissue or ethanol for disinfection, and after the skin dried, electrical stimulation with varying amplitudes, frequencies, pulse widths, and multiples was applied to the TA muscle by the FES stimulator. IMUs recorded the angle of dorsiflexion, and the maximum dorsiflexion angle under different parameters was displayed after averaging (*n *= 6). Among them, healthy subjects might exhibit smaller standard deviations due to their smaller body size and age range (± 2 years), whereas FD patients have larger standard deviations.

In tests related to walking, such as sEMG acquisition, ankle data recording, switch tests, walking functional electrical stimulation, etc., both patients and normal subjects (sample sizes were six respectively) followed the training instructions. Throughout the testing process, each subject precisely walked 100 steps in a single trial, with brief pauses and direction changes allowed. All subjects were not instructed to perform deliberate exercises beforehand, such as maintaining the same step length and GCD.

During the fNIRS test, patients rested for over 15 min in a resting state to allow HbO to stabilize. In the task state, all patients were commended to complete the same task, which was to walk independently and continuously for ≈16 m (30 steps). The task state was followed by a resting state, and then the next task state continued.

In the wearing test, stickable and disposable hydro coagulation electrodes transmitted signals and fixed the sEMG sensor. Soft and disposable physiotherapy electrodes with a snap‐on design conducted the electric pulse and secured the FES stimulator. Patients could put on and take off the CI‐FES system, including the FES stimulator and RF sEMG sensor located at the affected side, and TA sEMG sensor at the unaffected side, within 1 min. Both types of electrodes were flexible and breathable, ensuring comfortable long‐term wear.

During foot drop treatment, the adjusted FES was added to patients’ treatment courses. Patients wore the CI‐FES system, wiping the skin at hydrocoagulation electrodes and physiotherapy electrodes with alcohol. The FD patients selected for comparison exhibited comparable physical conditions and followed similar treatment protocols. The variation introduced was the add or omit CI‐FES system, while both patients underwent traditional treatments such as physical therapy (involving independent walking, squatting, and pedaling bicycles) and occupational therapy (covering self‐care, leisure, and productivity). The treatment involved 20 min of walking per day (including the unavoidable short breaks in between) for six days per treatment course. After each course, the patient's ankle angle was recorded, and evaluations were conducted using the FMA, Brunnstrom, and Ashworth scales (issued by the hospital). Due to the oil and sweat on the skin surface, the adhesiveness and conductivity of the hydrogel will decrease, and new hydrocoagulation electrodes and physiotherapy electrodes will be replaced each time. These disposable, low‐cost electrodes ensured the hygiene and efficacy of the treatment.

### IMU Sensor Testing and Data Analysis

The inertial measurement unit used was a high‐performance, compact, low‐latency 9‐axis wireless attitude sensor produced by HiPUC (HI221). Given that the IMU data was referenced to its own frame, the posture during measurement was crucial. All IMUs were positioned uniformly and secured as depicted in Figure S22 (above is the IMU_up_, below is the IMU_down_, Supporting Information). In a stationary stance, two IMUs with identical postures represent two coordinate systems in the same posture but in different positions. At this point, the angle between the coordinate systems was 0°, and the ankle joint angle was 90°, denoting the prestored angle as 90°. The back‐and‐forth swing of the calf, and the lifting‐and‐lowering of the toes during walking alter the pre‐stored angle. Consequently, the relative angle between ankle joints is calculated as follows:

(1) $$\mathrm{Ankle}\;\mathrm{angle}={\mathrm{IMU}}_{\mathrm{up}}+{\mathrm{IMU}}_{\mathrm{down}}+90^{\circ }$$

The coordinate transformation of the angular velocity adheres to a similar principle as detailed above (Note S4, Supporting Information).

### FNIRS Testing and Hemo Analysis

The assessment of brain function activation utilized the NirSmart‐6000, a portable, full‐coverage (over 60 channels), wireless, and highly stable produced by HuiChuang. The testing involved a 16 m distance after a resting period, with an active movement mode. NirSpark, the accompanying analysis software, allowed for customizable 3D models that varied based on the test probe channel. During data processing, spline interpolation to address motion artifacts was used, while physiological noise from heartbeat and respiration underwent 0.01–0.2 Hz band‐pass filtering. The filtered optical density data was transformed into changes in concentration of HbO, HbR, and HbT based on the modified Beer‐Lambert law. Following calculations using block averaging and the constructed model, effective segmented data was derived and visualized in the 3D diagram within the software and depicted in Figure 4i.

### Analytical Models for CI‐FES System Therapy

The SVM‐supervised learning algorithm for artificial intelligence analysis was used, which has a high recognition rate of 100% for FD patients and normal subjects, and has a robust ability to assess the recovery effect. Data collected underwent screening and segmentation to acquire 100 gait samples during patient walking. Subsequently, the maximum and minimum ankle joint angles, maximum angular velocity, and gait duration of the left and right feet were extracted for these samples to form a feature matrix. These calculated features were divided into ten parts, with each part serving as a validation set, while the remaining nine parts with labels act as the training sets and were input into the SVM model for training. The accuracy of the algorithm was evaluated by the average of 10 validation results (correct rate or error rate). To ensure the accuracy and consistency of the results, all data analysis methods during testing and treatment employed the SVM model with median Gaussian kernel function and ten‐fold cross‐validation. The program for eigenvalue matrix extraction, as well as the establishment and analysis of the SVM model, was all scripted in MATLAB 2020a.

### Statistical Analysis

The sEMG signal transmitted via WiFi was printed through the serial port and displayed directly after saving. To assess the RF muscle sEMG voltage and conduct feasibility statistics, the Findpeaks function in MATLAB was employed, capturing activated sEMG signals in 100 gaits. The results are presented in Figure 2d, with a statistical interval of 0.05 V. In evaluating the accuracy of the activation switch, threshold judgment procedures were applied in MATLAB, with the inclination judgment set at −25°, foot pressure at 0.25 V, and myoelectricity at 0.2 V. The statistical results were averaged (mean ± SD, *n *= 3) and displayed in Figure 2f and Figure S11 (Supporting Information). During the exploration of FES parameters, the displayed angle represents the maximum change in ankle dorsiflexion induced by FES (mean ± SD, *n *= 6), angular velocity was calculated as the average within the dorsiflexion interval of the ankle joint, (mean ± SD, *n *= 6). The Gaussian distribution curve characterizing the ankle joint was constructed with an interval of 5° on the distribution diagram. The mean value of the distribution curve signifies central tendency, while the variance represents the discrete trend. In practical calculations, the ankle joint angle curve generated during gait utilizes the Findpeaks function to extract values and positions of peaks and troughs (MaxAA and MinAA). Multiplying the number of points between the two peaks by the period yields the GCD. The same peak search function was applied to the angular velocity curve in gait to analyze maximum angular velocity. The feature values measured during patients’ CI‐FES treatment were inputted into the MATLAB machine‐learning application. Accuracy and receiver operating characteristic curves were then analyzed according to the mentioned configurations. The motor performance scale presents the mode (*n *= 4, without decimal precision, and mean ± SD not applicable), utilizing ANOVA to scrutinize differences in the before‐and‐after comparison, and Bonferroni correction was used to correct for multiple comparisons. All data curves, scatter plots, and distribution charts were drawn using Origin2020.

## Conflict of Interest

The authors declare no conflict of interest.

## Author Contributions

S.X., C.L., and C.W. contributed equally to this work. W.T., J.L., and S.X. conceived of and designed the overall experiments. S.X. and C.L. conducted experiments and collected related data. S.X. and C.L. were responsible for manufacturing the hardware of the sEMG sensor and FES stimulator. S.X. and S. S. co‐develop the procedure of CI‐FES system. S.X., C.W., X.K., and J. L. jointly conduct clinical tests at The Second Affiliated Hospital of Nanchang University. S.X., G.L., Z.X., and M.H. analyzed data from laboratory tests and clinical tests. S.X. and W.T. co‐wrote the paper. W.T. and J.L. supervised the project. All authors discussed the results and commented on the manuscript.

## Supporting information

Supporting Information

Supplemental Movie 1

Supplemental Movie 2

Supplemental Movie 3

Supplemental Movie 4

## Data Availability

The data that support the findings of this study are available from the corresponding author upon reasonable request.
